# 366. Antibiotic Stewardship Program Improved the Susceptibility of Commonly Seen Gram-negative Bacilli Over 12 Years at Singapore General Hospital

**DOI:** 10.1093/ofid/ofae631.107

**Published:** 2025-01-29

**Authors:** Peijun Yvonne Zhou, Yi Xin Liew, Shena Yun Chun Lim, YiBo Wang, Winnie Lee, Nathalie Chua, Li Wen Loo, Lai Wei Lee, Daphne Yah Chieh Yii, Narendran Koomanan, Kai Chee Hung, Jia Le Lim, Boon San Teoh, Jun Jie Tan, Li Xuan Trevina Lee, Cherie Si Le Gan, Siew Yee Thien, Benjamin Pei Zhi Cherng, Maciej Piotr Chlebicki, Shimin Jasmine Chung, Lay Hoon Andrea Kwa

**Affiliations:** Singapore General Hospital, Singapore; Singapore General Hospital, Singapore; Singapore General Hospital, Singapore; Singapore General Hospital, Singapore; Singapore General Hospital, Singapore; Singapore General Hospital, Singapore; Singapore General Hospital, Singapore; Singapore General Hospital, Singapore; Singapore General Hospital, Singapore; Singapore General Hospital, Singapore; Singapore General Hospital, Singapore; Singapore General Hospital, Singapore; Singapore General Hospital, Singapore; Singapore General Hospital, Singapore; Singapore General Hospital, Singapore; Singapore General Hospital, Singapore; Singapore General Hospital, Singapore; Singapore General Hospital, Singapore; Singapore General Hospital, Singapore; Singapore General Hospital, Singapore; Singapore General Hospital, Singapore

## Abstract

**Background:**

Antibiotic stewardship program (ASP) is advocated in all healthcare institutions to combat rising bacteria resistance. In 2008, ASP was established at Singapore General Hospital, with strategies focused on promoting appropriate use of anti-pseudomonal agents at first and expanding to ceftriaxone in 2015. Strategies include: 1) prospective audit feedback (PAF); 2) antibiotic guidelines; 3) digital solutions (computerized decision support system & mobile application); and 4) physician education [Figure 1]. In this study, we aim to evaluate the susceptibility rates of common gram-negative bacilli (GNB) and appropriate antibiotic use over time with ASP implementation.

**Figure 1:**
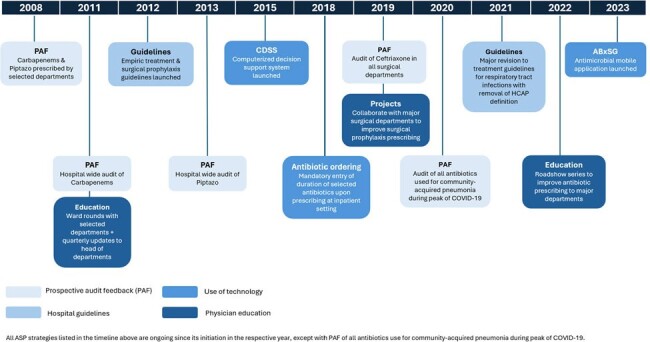
Antibiotic stewardship strategies implemented at Singapore General Hospital from 2008 to 2023

**Methods:**

We conducted an observational study from 2011 to 2023. Kendall’s tau coefficient (τ) was used to correlate the trend of bacteria susceptibility and percentage of appropriate antibiotic use with time. Susceptibility results of *E.coli* (EC), *Klebsiella species* (excluding *Klebsiella aerogenes), Pseudomonas aeruginosa* (PA) and *Acinetobacter baumannii* (AB) towards ceftriaxone (CRO), co-amoxiclav (AMC), piperacillin-tazobactam (PT), ertapenem (ERT) and meropenem (MEM), where applicable, was established based on breakpoints by Clinical and Laboratory Standards Institute. Percentage of appropriate MEM, ERT and PT use were derived from hospital-wide PAF.

**Figure 2:**
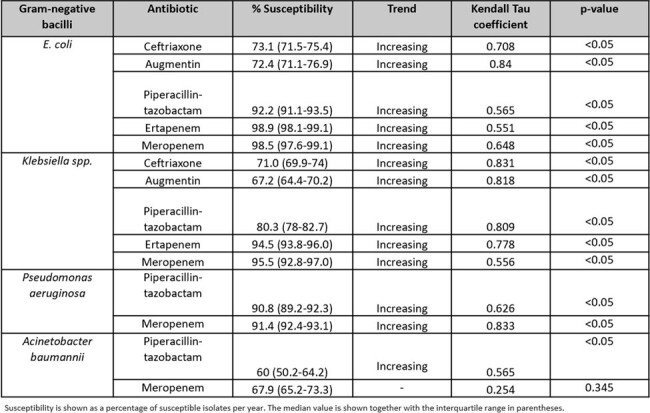
Trends of antibiotic susceptibility of E. coli, Klebsiella spp., Ps. aeruginosa and A. baumannii at Singapore General Hospital (2011-2023)

**Results:**

Susceptibility of EC and *Klebsiella spp*. towards CRO, AMC, PT, ERT and MEM have significantly increased over time (p < 0.05). Susceptibility of PA and AB towards MEM have also significantly increased (p < 0.05) [Figure 2, 3].

Notably, % of appropriate MEM and ERT use from 2011 to 2023 have significantly improved from 72.7% to 85.9% (τ= 0692, p < 0.05) and 75.9% to 92.0% (τ= 0.72, p < 0.05) respectively [Figure 4]. Percentage of appropriate PT use from 2013 to 2023 increased from 72% to 80.4% (τ= 0.055, p= 0.815).

**Figure 3:**
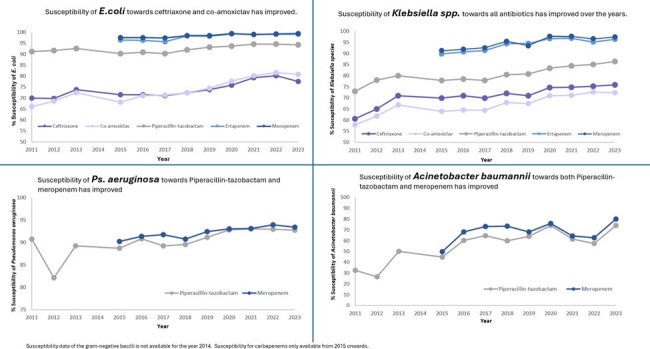
12- year trend of the susceptibility of Gram-negative bacilli towards commonly used broad-spectrum antibiotics from 2011 to 2023

**Conclusion:**

ASP is pertinent in promoting appropriate antibiotic use through multi-pronged approaches. Importantly, ASP’s goal of improving susceptibility of common GNB towards several broad-spectrum antibiotics was achieved. With increasing appropriate carbapenem use reaching a saturable stage, ASP must remain adaptable and design innovative strategies to expand its reach to even narrower spectrum antibiotics.

**Figure 4:**
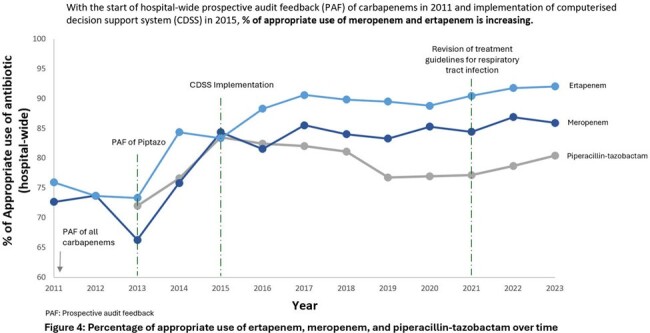
Percentage of appropriate use of ertapenem, meropenem, and piperacillin-tazobactam over time

**Disclosures:**

**All Authors**: No reported disclosures

